# Desialylated Platelet Clearance in the Liver is a Novel Mechanism of Systemic Immunosuppression

**DOI:** 10.34133/research.0236

**Published:** 2023-10-05

**Authors:** June Li, Danielle Karakas, Feng Xue, Yingyu Chen, Guangheng Zhu, Yeni H. Yucel, Sonya A. MacParland, Haibo Zhang, John W. Semple, John Freedman, Qizhen Shi, Heyu Ni

**Affiliations:** ^1^ Department of Laboratory Medicine and Pathobiology, University of Toronto, Toronto, ON, Canada.; ^2^ Toronto Platelet Immunobiology Group, Toronto, ON, Canada.; ^3^ Keenan Research Centre for Biomedical Science of St. Michael’s Hospital, Toronto, ON, Canada.; ^4^ Canadian Blood Services Centre for Innovation, Toronto, ON, Canada.; ^5^ Departments of Pediatrics, Medical College of Wisconsin, Milwaukee, WI, USA.; ^6^ Blood Research Institute, Versiti Wisconsin, Milwaukee, WI, USA.; ^7^ Departments of Ophthalmology and Vision Sciences Medicine, University of Toronto, Toronto, ON, Canada.; ^8^ Faculty of Engineering and Architectural Science, Ryerson University, Toronto, ON, Canada.; ^9^ Multi-Organ Transplant Program, Toronto General Hospital Research Institute, Toronto, ON, Canada.; ^10^ Department of Immunology, University of Toronto, Toronto, ON, Canada.; ^11^ Critical Care Medicine, Department of Anesthesiology and Pain, University of Toronto, Toronto, ON, Canada.; ^12^ Department of Physiology, University of Toronto, Toronto, ON, Canada.; ^13^ Department of Pharmacology, University of Toronto, Toronto, ON, Canada.; ^14^ Division of Hematology and Transfusion Medicine, Lund University, Lund, Sweden.; ^15^ Clinical Immunology and Transfusion Medicine, Office of Medical Services, Region Skåne, Lund, Sweden.; ^16^ Department of Medicine, University of Toronto, Toronto, ON, Canada.; ^17^ Children’s Research Institute, Children’s Wisconsin, Wauwatosa, WI, USA.; ^18^ Midwest Athletes Against Childhood Cancer Fund Research Center, Milwaukee, WI, USA.

## Abstract

Platelets are small, versatile blood cells that are critical for hemostasis/thrombosis. Local platelet accumulation is a known contributor to proinflammation in various disease states. However, the anti-inflammatory/immunosuppressive potential of platelets has been poorly explored. Here, we uncovered, unexpectedly, desialylated platelets (dPLTs) down-regulated immune responses against both platelet-associated and -independent antigen challenges. Utilizing multispectral photoacoustic tomography, we tracked dPLT trafficking to gut vasculature and an exclusive Kupffer cell-mediated dPLT clearance in the liver, a process that we identified to be synergistically dependent on platelet glycoprotein Ibα and hepatic Ashwell–Morell receptor. Mechanistically, Kupffer cell clearance of dPLT potentiated a systemic immunosuppressive state with increased anti-inflammatory cytokines and circulating CD4^+^ regulatory T cells, abolishable by Kupffer cell depletion. Last, in a clinically relevant model of hemophilia A, presensitization with dPLT attenuated anti-factor VIII antibody production after factor VIII ( infusion. As platelet desialylation commonly occurs in daily-aged and activated platelets, these findings open new avenues toward understanding immune homeostasis and potentiate the therapeutic potential of dPLT and engineered dPLT transfusions in controlling autoimmune and alloimmune diseases.

## Introduction

Platelets are small blood cells generated from megakaryocytes, are abundant in the blood, and are well-known essential contributors to hemostasis and thrombosis [1–5]. Platelets also contain a plethora of immune modulators and receptors, giving way to a growing appreciation for them as critical sentinels of immunity [6–8]. It has been well reported that platelets act as integral players in various stages of innate immunity and can help shape adaptive immune responses [6,8,9]. Acting through either direct contact with leukocytes [10] or secreted factors including microparticles [11], platelets contribute to the propagation of proinflammation in various diseases [12–14], cementing their status as an essential link within the thrombo-immuno-axis [15]. Despite their well-recognized role in proinflammation, the anti-inflammatory/immunosuppressive role of platelets is not well explored.

Platelets constantly undergo removal from the bloodstream following normal senescence, activation, or in disease states. The most common signals for active platelet clearance include exposure or binding of key molecules such as phosphatidylserine, P-selectin, or in a pathological context of antibody binding [16–19]. This leads to canonical clearance pathways within the reticuloendothelial system of the spleen. One unique mechanism by which platelets regulate self-clearance is the endogenous modification of surface glycans [20]. In particular, desialylation, the removal of platelet terminal sialic residues, leads to increased trafficking and clearance of desialylated platelets (dPLTs) to the liver [21,22]. This has recently been shown to be a dominant process during platelet aging [23]. We and others have previously demonstrated that premature desialylation could also be induced via activating signals from antibody, CD8^+^ cytotoxic T lymphocytes or microbial binding [22–27]. Antibody and cytotoxic T lymphocyte-induced platelet desialylation and subsequently clearance in the liver may play important role in immune thrombocytopenia that can be ameliorated by sialidase inhibitors such as oseltamivir [22,23,27–29]. However, the receptors that dictate platelet targeting to the liver remain not well defined. More importantly, the systemic implications of dPLT clearance within the unique immunotolerant niche of the liver have never been explored.

Here, we observe that dPLT clearance in the liver stimulates an antigen-independent systemic immunosuppressive response whereby a secondary challenge of platelets or sheep red blood cells (sRBCs) results in lower antibody generation. We attribute this phenomenon to dPLT clearance via hepatic Kupffer cells and identify that this process is synergistically mediated by both platelet glycoprotein Ibα (GPIbα) and the Ashwell–Morell receptor (AMR). Immunological sequelae of Kupffer cell dPLT uptake included increased anti-inflammatory cytokine production and circulating regulatory T cells (T_regs_), which was attenuated following Kupffer cell depletion. To establish translational potential, we further tested a clinically relevant murine model of hemophilia A [factor VIII (FVIII)-deficient, FVIII^null^] and found that presensitization with dPLT significantly attenuated anti-FVIII inhibitory antibody (inhibitor) generation in FVIII^null^ mice following recombinant human FVIII (rhFVIII) transfusion. These findings introduce a novel anti-inflammatory role of platelets that contrasts the prevailing proinflammatory view. It elucidates a previously unknown mechanism by which platelets contribute to the maintenance of immune quiescence and opens new therapeutic avenues for the utilization of dPLTs to control alloimmune and autoimmune diseases [30–32].

## Results

### dPLTs induce immunosuppression

In many disease states, platelet surface antigens, particularly GPIIbIIIa (integrin αIIbβ3) and GPIbα, become immunogenic and are frequent targets in immune-mediated thrombocytopenias [17,33–36]. Platelets are heavily and dynamically glycosylated, and desialylation frequently occurs in circulation due to pathological or normal homeostatic processes such as senescence [8,20]. We hypothesized that removal of terminal sialic acids could enhance antigen presentation and induce a more robust immune response [37,38]. To test this, we transfused wild-type (WT) or desialylated WT platelets (dPLT) into GPIbα^−/−^ or β3^−/−^ mice to induce an adaptive immunoglobulin G (IgG) immune response against platelet GPIbα or β3, respectively. Prior to transfusion, it was confirmed that ex vivo desialylation did not cause shedding of GPIbα or β3 antigen (Fig. S1).

**Fig. 1.**
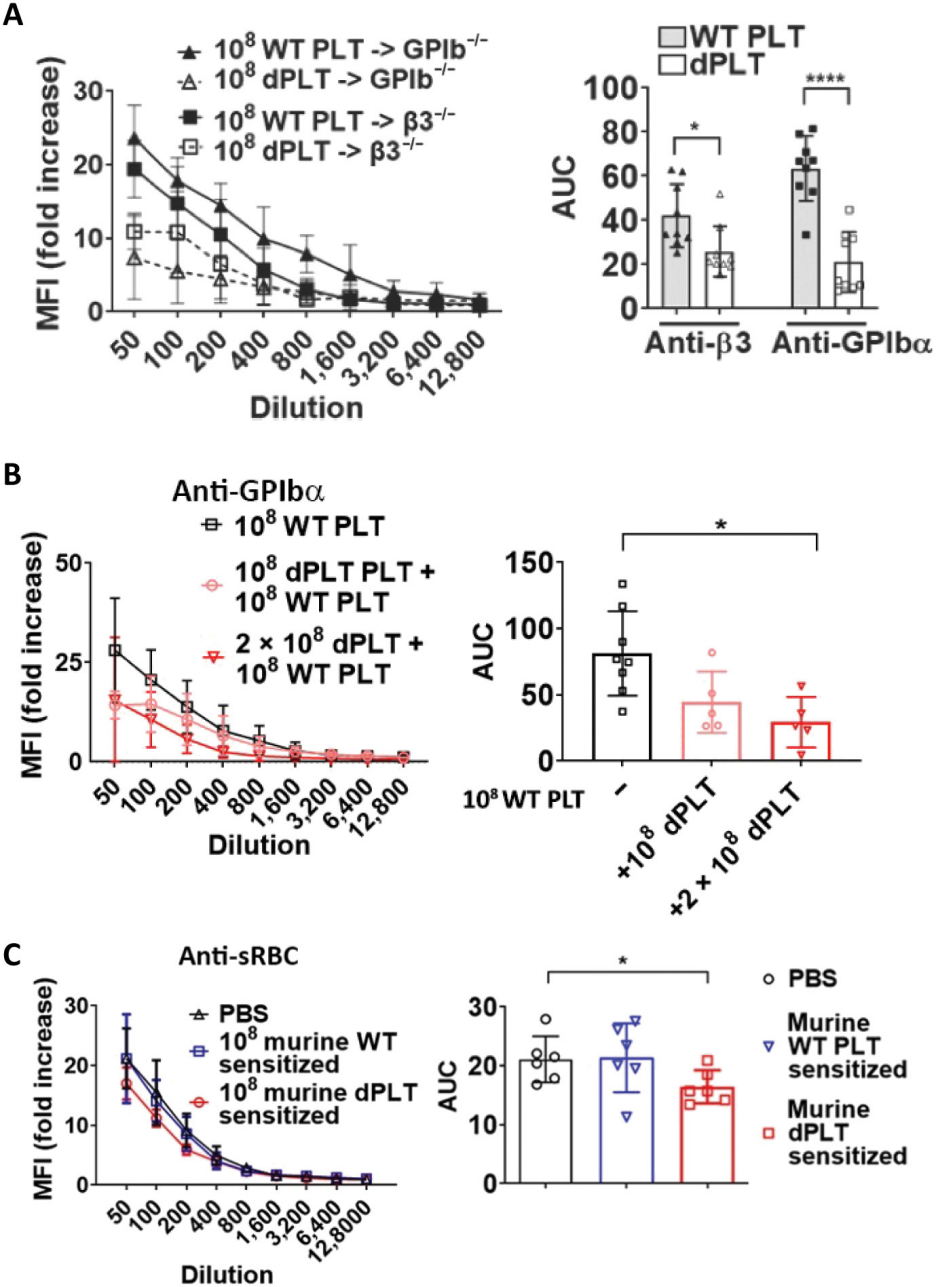
dPLTs induce immunosuppression. (A) Antibody titers in GPIbα^−/−^ and β3^−/−^ mice intravenously immunized with 10^8^ WT platelets (WT PLT) or dPLTs (dPLT). (B) Antibody titers in GPIbα^−/−^ mice cocurrently intravenously immunized with 10^8^ WT platelets with increasing doses of dPLTs. (C) Anti-sRBC antibody titers in WT BALB/c mice preinjected with 2 × 10^8^ WT or dPLTs. Statistical analysis was done with one-way analysis of variance (ANOVA) with Tukey post hoc test. All data represented as means ± SD and quantified as area under the curve (AUC). *P < 0.05 and ****P < 0.001.

Contrary to expectations, we observed significantly lower antibody titers when dPLTs were transfused, particularly in GPIbα^−/−^ mice (Fig. 1A). Interestingly, when sRBCs were transfused as an immunogen, there was no significant difference in anti-sRBC IgG production between desialylated versus nondesialylated sRBC (Fig. S2), suggesting that this lower antibody response is a platelet-specific effect. As antibody titers were detected through binding of serum IgG to WT non-dPLTs, we also tested the binding of IgG to dPLT and saw consistent lower antibody titers (Fig. S3). This excludes that the observed lower antibody response was due to the generation of a distinct antibody repertoire to novel epitopes following desialylation.

To assess whether dPLTs were merely less immunogenic or could immunomodulate a response, we next cotransfused dPLT with same amounts of WT platelets and found that the presence of dPLT decreased antibody response dose dependently (Fig. 1B). To test whether the immunosuppressive effect was specific to platelet antigens or is broadly immune dampening, we presensitized WT mice with a desialylated or WT platelet transfusion, followed by a nonplatelet antigen challenge with sRBCs. We observed a slight, albeit significant decrease in anti-sRBC titers in mice pretransfused with dPLT (Fig. 1C). These data indicate that clearance of dPLT in vivo may lead to an anti-inflammatory and immunosuppressive state that dampens a cocurrent immune response.

### dPLTs target to the gut vasculature at early time points and are exclusively cleared in the liver

**Fig. 2.**
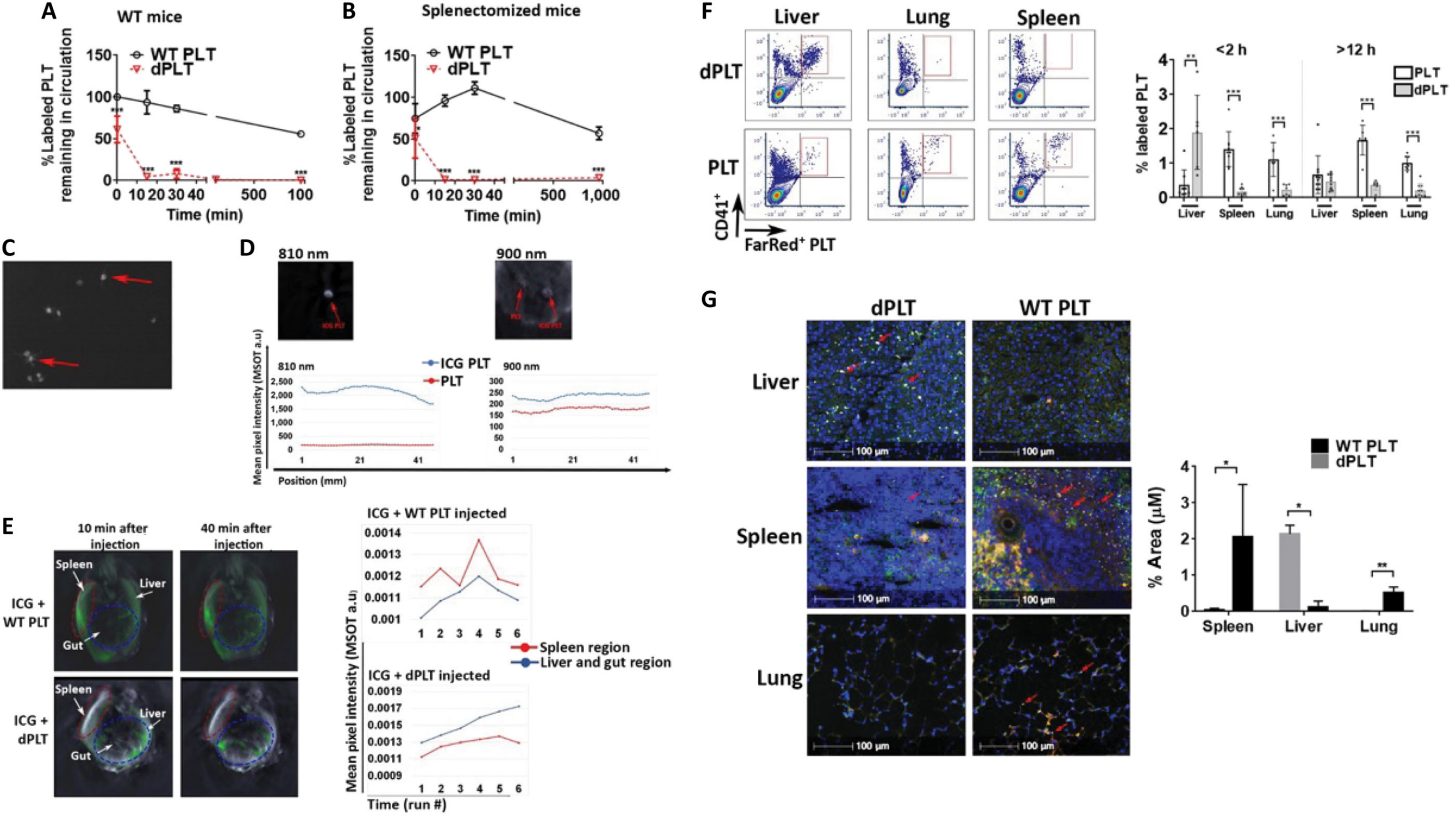
dPLTs target to the gut vasculature at early time points and are exclusively cleared in the liver. (A and B) CellTracker-labeled WT platelets or dPLTs were intravenously transfused into (A) WT or (B) splenectomized mice. At 15 min, 30 min, 2 h, and 16 h after injection, remaining labeled platelets in circulation was assessed by flow cytometry and calculated as percentage of baseline (percent in circulation at 1 min after injection). *n* = 10 per group. (C) Representative immunofluorescence images of ICG-labeled platelets. Arrows point to labeled platelets. (D) Representative images of MSOT scans of ICG-labeled platelets and unlabeled platelets in agar phantoms. Tracings represent mean optoacoustic intensities across the length of the agar phantom at nonspecific 900-nm and ICG-specific 810-nm excitation wavelengths. (E) Representative 3D reconstructed images of MSOT scans over 40 min of mice transfused with ICG-labeled dPLTs and platelets. Tracings represent mean optoacoustic intensities across 60 min of 2 regions in mice. Blue line, the liver and gut region; red line, spleen. (F) Representative dot plots and quantification of CellTracker^+^CD41^+^ platelets in indicated organs at early (<2 h) and late (>12 h) time points. Data represented as means ± SD. *P < 0.05, **P < 0.01, and ***P < 0.001. (G) Representative immunofluorescence of CellTracker^+^ platelets in various organs at 2 h after intravenous transfusion. Bar graph is quantification of % area of whole tissue section positive for CellTracker^+^ platelets as assessed by HALO software. White, CellTracker^+^ platelets; green, F4/80^+^ macrophages; blue, DAPI. Arrows point to localized CellTracker^+^ platelet.

The observed immunosuppressive effects following dPLTs transfusion may be attributable to the local clearance mechanisms and immunological niche responses. Although we and others have previously demonstrated that dPLTs are cleared in the liver [8,22,39–41], other contributory organs have not been well explored, and we thus investigated the biodistribution of dPLT clearance. dPLTs were fluorescently labeled ex vivo to allow for in vivo tracking via flow cytometry. Following intravenous transfusion, venous blood sampling at various time points indicated dPLTs exhibited rapid clearance kinetics similar to what was previously reported [22,42] (Fig. 2A). Unexpectedly, this process was completely independent of the spleen as we did not observe a significant rescue in dPLT clearance in splenectomized mice (Fig. 2B).

To determine the organs of acute dPLT clearance, we developed a novel technique utilizing photoacoustic tomography (PAT) to noninvasively assess dPLT real-time clearance mechanisms in vivo, circumventing potential post-mortem artifacts of nonspecific blood pooling. PAT photonically excites tissue with wavelengths in the near-infrared red region (>700 nm), allowing for deep tissue penetration, minimal photon light scattering, and detection of the signal by an ultrasound transducer with ultrasound resolution [43]. Multispectral unmixing, known as multispectral optoacoustic tomography (MSOT), further allows for tracking target biomolecules such as transfused platelets when coupled to specialized contrast agents with distinct absorption spectra [44]. We utilized a well-characterized Food and Drug Administration-approved indocyanine green (ICG), commonly used as a contrast agent in humans [45], to couple to platelets. To the best of our knowledge, we are the first to utilize PAT to track platelet biodistribution in real time, in vivo. Confocal microscopy revealed platelet uptake of ICG when incubated together (Fig. 2C). Spectrometric measurement of absorbance of ICG-labeled platelets every 10 nm from 600 to 900 nm showed maximum absorbance between 800 and 810 nm, consistent with previous published spectral values of ICG coupled to biomolecules (Fig. S4) [46]. Preliminary assessment in MSOT with phantom agar molds was used to test limits of detection and signal strength in relation to the concentration of dPLT. We found at low concentrations of 10 [6] ICG-labeled platelets, the signal was ~3× that of unlabeled platelets at an ICG-maximum absorption wavelength of 810 nm (Fig. 2D). We next transitioned to in vivo mouse MSOT scans of intravenously transfused with ICG-labeled WT platelet or dPLT. We observed a rapid increase in signal accumulation in the liver and gut vasculature of ICG-labeled dPLT, with a gradual decrease in signal in the spleen over the course of 60 min. Whereas control ICG-labeled platelets continue to circulate throughout the mice (Fig. 2E). These kinetics are consistent with the rapid disappearance of dPLT from circulation as observed by flow cytometry (Fig. 2A). To the best of our knowledge, dPLT localization to the gut has not been previously reported. To confirm, we further utilized intravital microscopy [47–49] and observed dPLT stable adherence to mesenteric vasculature (Fig. S5). Furthermore, small amounts of fluorescently labeled dPLT were detectable via flow cytometry and tissue immunofluorescence of the small intestine, suggesting potential translocation of dPLT to underlying gut-associated lymphoid tissue (Figs. S6 and S7)

We further harvested different organs to track platelet accumulation with flow cytometry at various time points. We observed only a significant accumulation of dPLT in the liver of mice at early time points (<2 h) (Fig. 2F), but not at late time points (>12 h), indicating dPLT clearance. Transfused control platelets were predominantly localized to the spleen and lung at both the early and late (>12 h) time points, which, concomitant with its sustained presence in circulation, suggests nonspecific pooling during organ collection. It is noteworthy that there was scant accumulation of dPLT in the lung (Fig. 2G) despite its anatomical positioning as the first large vascular bed following intravenous tail–vein transfusion. This indicates that dPLT are actively sequestered in the liver, rather than passive adherence within the vasculature.

### Platelet hepatic targeting requires synergistic platelet GPIbα and hepatic AMR

**Fig. 3.**
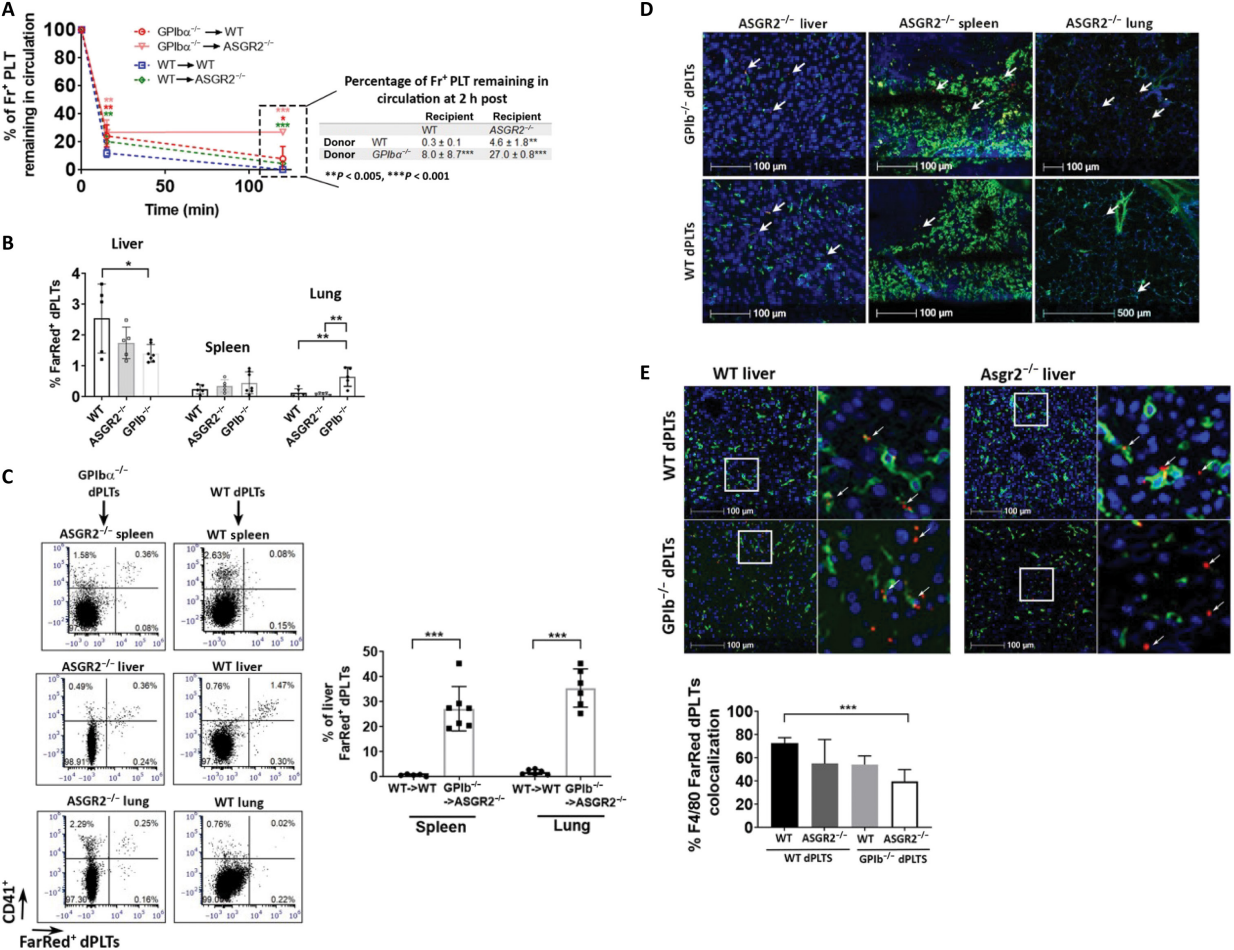
Platelet hepatic targeting requires synergistic platelet GPIbα and hepatic AMR. (A) Percentage of CellTracker-labeled desialylated WT or GPIbα^−/−^ platelets remaining in circulation at time of 15-min and 2-h time points following intravenous transfusion into WT of ASGR2^−/−^ mice. Data shown as percentage of baseline (1 min after injection). Significant data points are compared to WT. *n* = 5 to 9. (B and C) Percentage of FarRed^+^ dPLTs as determined by flow cytometry within organs at 2 h after intravenous transfusion. *n* = 5 to 6 per group. (C) Percentage represented as (*x* / % FarRed^+^ dPLTs in the liver × 100). *n* = 6 to 8 per group (D and E) Representative immunofluorescence images of indicated organs harvested at 2 h after intravenous transfusion of FarRed^+^ dPLTs. Bar graph is % area of FarRed^+^ dPLT that is also positive for F4/80^+^. Green, F4/80^+^ macrophages; red, FarRed^+^ dPLTs; blue, DAPI. Arrows point to FarRed^+^ dPLTs. Statistical analysis was done by one-way ANOVA with Tukey post hoc, *P < 0.05, **P < 0.01, and ***P < 0.001.

Mechanisms that exclusively target dPLT to the liver are not well understood. Previously, the hepatic AMR was implicated as a critical receptor in sequestration and uptake of dPLT in the liver [21,22]. We have previously demonstrated platelet GPIbα is required for platelet-mediated hepatic thrombopoietin production [50,51], suggesting that both receptors actively regulate hepatic dPLT targeting. To elucidate the contributory role of each receptor, we assessed dPLT clearance in AMR deficient (*ASGR2*^−/−^) mice and with GPIbα^−/−^ platelets. We found that in *ASGR2*^−/−^ mice, transfusion of CellTracker-labeled WT dPLT resulted in minimal but significant rescue of dPLT clearance at 2 h after injection (mean, 4.6 ± 1.8 versus 0.3 ± 0.1) (Fig. 3A). Similarly, when CellTracker-labeled GPIbα^−/−^ dPLTs were transfused into WT recipients, rescue of dPLT clearance from circulation was higher than in *ASGR2^−/−^* mice and significantly different from WT dPLT transfusion (mean, 8.0 ± 8.7 versus 0.3 ± 0.1). Interestingly, lack of both AMR and GPIbα (GPIbα^−/−^ dPLTs into *ASGR2^−/−^* mice) resulted in a synergistic effect, with clearance of dPLT plateauing at the 15-min mark (mean, 27.0 ± 0.8). Despite the significant rescue, the majority of GPIbα^−/−^ dPLT (~75%) remains cleared from circulation (Fig. 3A), suggesting that other mechanisms might be involved although the roles of αIIbβ3 and/or other antigens in this process are still unclear. Further investigation into sequestering organs revealed a predominantly hepatic localization in the absence of one of the receptors, although GPIbα^−/−^ platelets exhibited significant targeting to the lung at the 2-h mark (Fig. 3B). This suggests that neither AMR nor GPIbα alone is sufficient for dPLT hepatic targeting. However, when both receptors were absent (GPIbα^−/−^ dPLT into *ASGR2*^−/−^ mice), although the kinetics of platelet clearance were similar, the organ distribution was significantly altered. Specific targeting to the liver was abolished, and diffuse GPIbα^−/−^ dPLT localization in the *ASGR2*^−/−^ spleen and lung represented ~30% of dPLT in the liver (mean, 35.4 ± 7.6 and 27.2 ± 8.8, respectively) (Fig. 3C).

Immunofluorescence further confirmed significant GPIbα^−/−^ dPLT localization in the *ASGR2*^−/−^ spleen and lung (Fig. 3D). Moreover, colocalization with Kupffer cells was decreased in the absence of platelet GPIbα but not AMR alone (Fig. 3E). These data suggest that platelet GPIbα synergistically with the AMR mediates dPLT targeting to the liver with GPIbα-mediating interaction with Kupffer cells.

### Kupffer cells are required for dPLT clearance and are functionally distinct from splenic macrophages

**Fig. 4.**
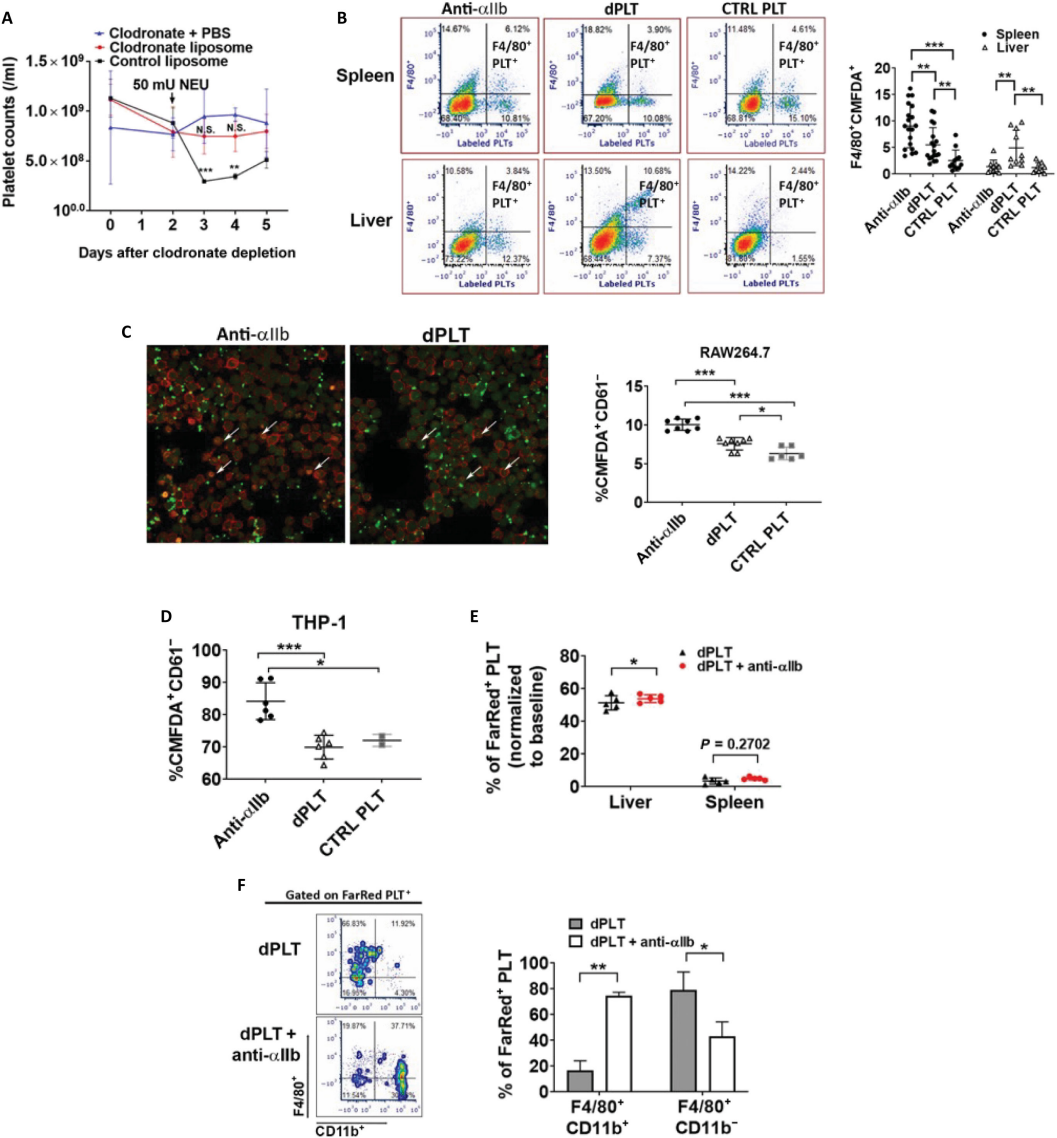
Kupffer cells are required for dPLT clearance and are functionally distinct from splenic macrophages. (A) Platelet counts of WT BALB/c mice intravenously transfused with NEU with or without Kupffer cell depletion. (B to D) In vitro coculture of (B) primary Kupffer and splenic macrophages, (C) RAW624.7, (D) differentiated human THP-1 with CellTracker^+^ WT (CTRL PLT), dPLT, or anti-αIIb-opsonized platelets. Macrophage-phagocytosed platelets are defied as (B) Kupffer F4/80^+^CellTracker^+^ platelets and (C and D) CellTracker^+^CD61^−^ platelets. *n* = 6 to 7 per group. Statistical analysis was done by one-way ANOVA with Tukey post hoc test. (C) Representative immunofluorescence images comparing phagocytosis of anti-αIIb-opsonized platelets or dPLT by RAW624.7 cells. Green, CellTracker^+^ platelet; red, F4/80^+^ macrophages. Arrows point to orange internalized platelets localized intracellularly. (E) Percentage of FarRed^+^ dPLTs as determined by flow cytometry within harvested organs at 1 h after intravenous transfusion. Data shown are normalized to % FarRed^+^ platelets in circulation at a time of 1 min after injection. *n* = 5. (F) Representative density plot and graph gated on FarRed^+^ platelets of showing predominant association of anti-αIIb-opsonized FarRed^+^ platelets with monocyte-derived CD11b^+^F4/80 and dPLTs with CD11b^lo^F4/80^+^ Kupffer. *n* = 5. *P < 0.05, **P < 0.01, and ***P < 0.001.

As we observed above, dPLTs were predominantly associated with macrophages in the liver; we hypothesized that Kupffer cells may be the central phagocytic cell to mediate dPLT clearance. To test this, we depleted macrophages with clodronate liposomes (Fig. S8), followed by injection of neuraminidase (NEU) to induce platelet desialylation in vivo. We found that thrombocytopenia was almost completely rescued in macrophage-depleted mice (Fig. 4A), suggesting almost complete attenuation of dPLT clearance.

We next transitioned to an in vitro culture system to assess the phagocytic potential of Kupffer cells in dPLT uptake. Utilizing single-cell suspensions from harvested livers, we measured via flow cytometry phagocytosis of fluorescently labeled dPLTs by CD11b^lo^F4/80^+^ Kupffer cells, which expressed litter/low levels of CD11b compared to monocyte-derived macrophages [52,53]. As expected, we observed significant phagocytosis of dPLT by Kupffer cells compared to non-dPLTs (Fig. 4B).

Interestingly, when antibody-opsonized platelets or splenic F4/80^+^ macrophages were used as a control, we observed that splenic macrophages were more proficient at engulfing antibody-opsonized platelets compared to dPLTs, with the inverse being true for liver macrophages (Fig. 4B). Similarly, both the murine splenic cell line RAW264.7 and the human monocytic THP-1 cell line exhibited preferential uptake of antibody-opsonized platelets compared with dPLTs (Fig. 4C and D). Thus, we are the first to show a functional bias between the 2 resident macrophages (i.e., liver versus spleen) for platelet clearance.

As antibody-opsonized platelets are predominantly cleared in the spleen, consistent with the preferential uptake by splenic macrophages in our in vitro culture system, we were curious as to whether antibody opsonization of dPLT would sufficiently abrogate hepatic targeting. Interestingly, when we injected CellTracker-labeled antibody-opsonized dPLT, it retained liver targeting (Fig. 4E). However, clearance is shifted to CD11b^+^ monocyte-derived macrophages and not Kupffer cells (Fig. 4F). These data suggest that Kupffer cells are specialized to clear dPLT, while monocyte-derived M1-type macrophages are functionally biased toward antibody-mediated clearance.

### Increased interleukin-10 and transforming growth factor-β production in the liver following Kupffer cell uptake of dPLTs

**Fig. 5.**
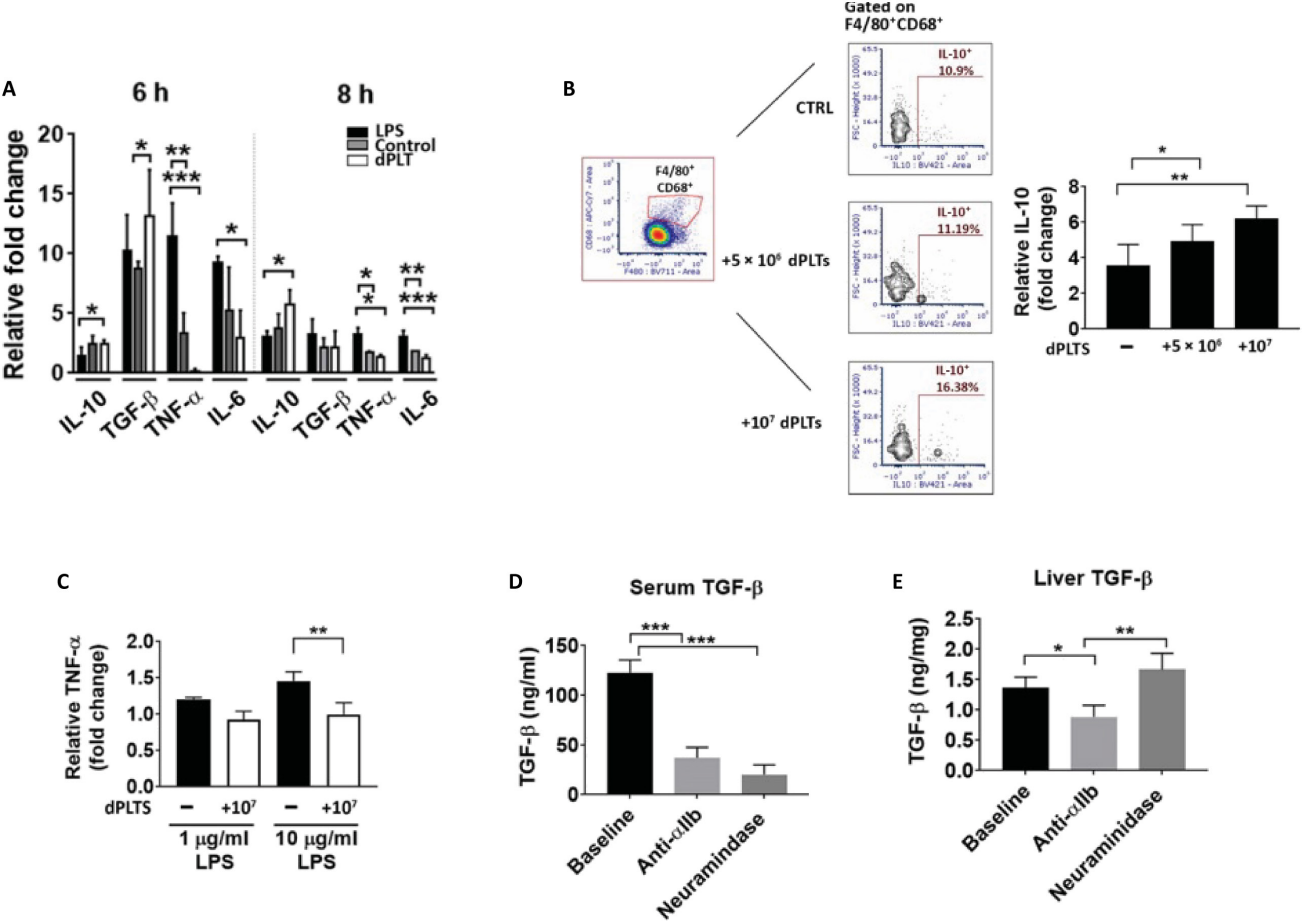
(A and B) In vitro analysis of cytokine changes by flow cytometry of primary Kupffer cells following coculture with dPLTs, LPS (10 μg/ml), or control (Kupffer cells alone). Data represented as fold change of % positive of F4/80^+^CD68^+^ cells per isotype control. *n* = 4 (duplicate). (C) Indicated concentration of LPS was added at 24 h after incubation with dPLT. *n* = 4 (duplicate). (D and E) Serum and liver TGF-β levels as measured by enzyme-linked immunosorbent assay day 1 after intravenous injection of 50 mU of NEU or anti-αIIb monoclonal antibody (0.2 μg/g). *n* = 6. Statistical analysis was done with one-way ANOVA with Tukey post hoc test. *P < 0.05, **P < 0.01, and ***P < 0.001.

Kupffer cells have been reported to contribute to the maintenance of the immunotolerant niche of the liver [54,55]. Since we have established that dPLTs are predominantly cleared by Kupffer cells, we next investigated the downstream immunological responses. In vitro coculture assays of dPLT with primary Kupffer cells revealed a dose-dependent increase in Kupffer cell production of interleukin-10 (IL-10) peaking at time of 8 h (Fig. 5A and B). Other Kupffer cell-derived cytokines produced small but nonsignificant increases (Fig. S9). We also observed a transient and variable increase in Kupffer cell-associated transforming growth factor-β (TGF-β) at 6 h after following dPLT coculture (Fig. 5A), which is likely derived from increased dPLT binding. To assess whether dPLT induction of increased IL-10 production polarizes Kupffer cells to a more immunosuppressive state, we added lipopolysaccharide (LPS) to Kupffer cell cultures following 24 h of coincubation with dPLT. We observed decreased production of proinflammatory tumor necrosis factor-α in Kupffer cells preincubated with dPLT (Fig. 5C) which suggests that dPLT promotes a shift toward anti-inflammatory IL-10 in Kupffer cells.

We next investigated that the direct effect platelet depletion would have on circulating and liver TGF-β levels. Anti-αIIb monoclonal antibody and NEU were injected to induce predominant splenic and hepatic clearance of platelets, respectively. Thrombocytopenia was confirmed to be similar in both injections (Fig. S10). On day 1 after injection, when platelet counts are at a nadir, serum TGF-β was found to be drastically decreased in both antibody- and NEU-injected mice (Fig. 5D). This is not unexpected as platelets in both cases are cleared from circulation and underscore the substantial contribution of platelets to circulating TGF-β levels. However, TGF-β levels in liver tissue were found to be significantly increased in NEU-injected mice but significantly decreased in antibody-injected mice (Fig. 5E) as dPLTs are accumulated to the liver, whereas antibody-opsonized platelets are routed to the spleen. Thus, dPLT targeting to the liver significantly contributes to local potent immunosuppressive TGF-β levels, which is abrogated when platelet clearance is diverted to the spleen.

### dPLT increases circulating and functional CD4^+^ T_regs_

**Fig. 6.**
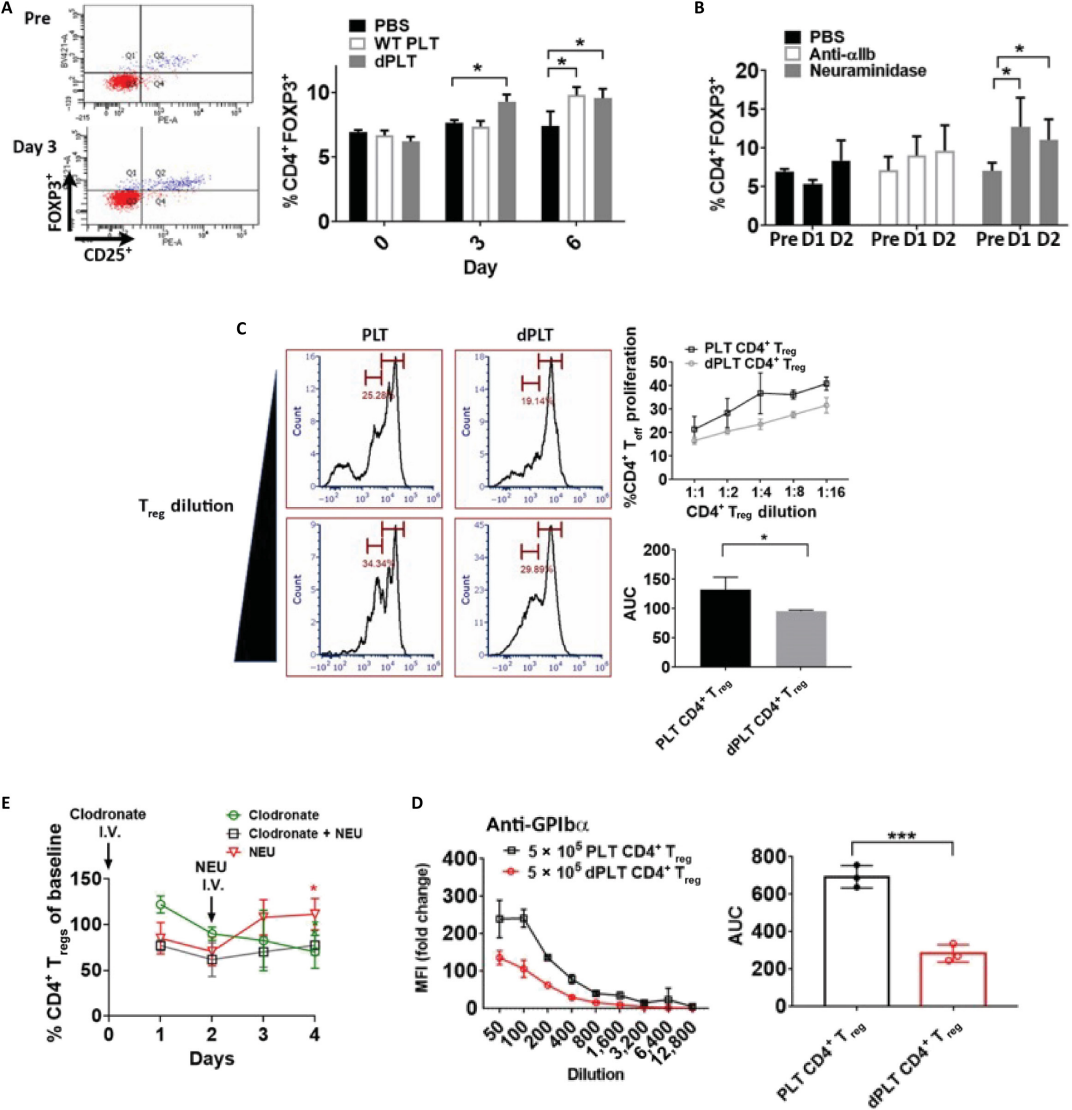
dPLT increases circulating and splenic functional CD4^+^ T_regs_. (A) Representative dot plot gated on CD4^+^ showing increased CD25^+^FOXP3^+^ T_regs_ in circulation on day 3 following intravenous transfusion with dPLTs. (A and B) Measurement of PBMC CD4^+^ T_regs_ on indicated days following intravenous transfusion of (A) 2 × 10^8^ dPLT or platelet (B) 50 mU of NEU or anti-αIIb antibody (0.02 μg/g). *n* = 6 per group. Statistical analysis done by with one-way ANOVA with Tukey post hoc test. (C) Graph and representative histogram of decreased CD4^+^ proliferation in vitro in presence of increasing dilution of splenic CD4^+^ T_regs_ isolated from desialylated or WT platelet transfused GPIbα^−/−^ mice. (D) Anti-GPIbα titers from 3× WT immunized GPIbα^−/−^ mice transfused with CD4^+^CD25^+^ splenic T_reg_ sorted from either dPLT- or WT platelet-treated mice. (E) Measurement of PBMC CD4^+^ T_regs_ on indicated days following macrophage depletion by clodronate liposomes. Data represented as percentage change from day 0 (immediately prior to clodronate injection). *n* = 4. *P < 0.05 and ***P < 0.001.

We next investigated whether increased production of immunosuppressive cytokines in the liver in the presence of dPLT clearance can drive a systemic response. We found that transfusion of dPLTs, or in vivo platelet desialylation with NEU, led to increased CD4^+^CD25^+^FOXP3^+^ T_regs_ in blood circulation (Fig. 6A and B) 3 days after injection. Transfusion of non-dPLTs also significantly induced circulating CD4^+^CD25^+^FOXP3^+^ at a later time point (~6 days). This could potentially be attributable to platelet aging that increased platelet desialylation and hepatic clearance. We next assessed whether the increased CD4^+^ T_regs_ possessed functional significance, specifically contributing to the lower immune response observed above (Fig. 1). We cell-sorted CD4^+^CD25^+^ splenic T cells following transfusion with dPLT or non-dPLT. An in vitro T cell proliferation assay revealed an enhanced suppressive function of CD4^+^ T cell proliferation with CD4^+^ T_regs_ isolated from dPLT compared to platelet-transfused mice (Fig. 6C). In vivo transfusion of CD4^+^ T_regs_ from dPLT-sensitized mice, followed by platelet challenge, led to a lower antibody response (Fig. 6D). Last, to assess the direct link with Kupffer cell clearance of dPLT and increased functional CD4^+^ T_reg_ generation, we depleted Kupffer cells with clodronate liposomes and measured circulating peripheral blood mononuclear cells (PBMCs). Previous reports demonstrate that Kupffer cells do not significantly repopulate by our experiment duration [56]. We found decreased circulating CD4^+^ T_regs_ at day 4 after injection (Fig. 6E), which could not be rescued by injection of NEU. This demonstrates that Kupffer cell contribution to CD4^+^ T_reg_ homeostasis is, in part, mediated by clearance of dPLT in the liver. Thus, through both anti-inflammatory/immunosuppressive cytokines and CD4^+^ T_regs_, dPLTs play a previously unexplored but important roles in maintenance of immune quiescence.

### dPLT infusion attenuates antibody generation in clinically relevant transfusion models

**Fig. 7.**
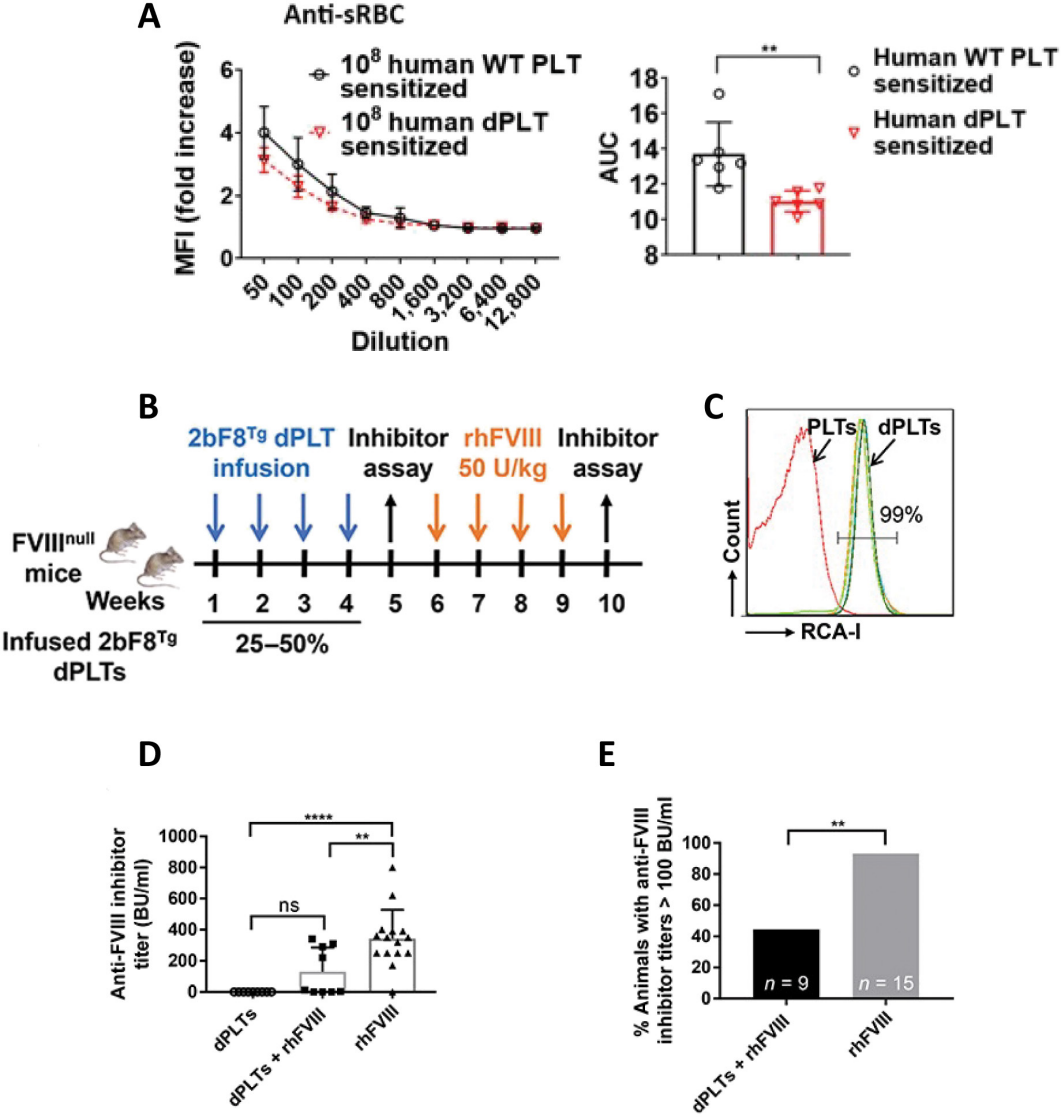
dPLT infusion attenuates antibody generation in clinically relevant transfusion models. (A) Anti-sRBC antibody titers in WT BALB/c mice preinjected 3 days prior with 10^8^ WT human dPLTs. Bar graphs show antibody titers quantified as AUC. *n* = 2 human donors and 3 mice per group. (B) Schematic diagram of the timeline of dPLT transfusion and FVIII immunization in naïve FVIII^null^ mice. (C) Flow cytometry analysis of dPLTs. Platelets without NEU treatment were used as a negative control. (D) Inhibitor titers of FVIII^null^ mice infused with desialylated 2bF8^Tg^ platelets followed by rhFVIII immunization (50 U/kg per week, ×4). FVIII^null^ mice without 2bF8^Tg^ dPLT infusion were used as a control in parallel. Statistically significant differences between the means of groups were determined by one-way ANOVA followed by Tukey’s multiple comparisons test. (E) Comparison of number animals that developed high inhibitory titers (>100 BU/ml) following rhFVIII immunization. Statistical significance was determined by Pearson’s test. **P < 0.01 and ****P < 0.0001. “n.s.” indicates no statistically significant difference between the 2 groups.

Transfusion-mediated immunogenic antibody reactions can lead to life-threatening adverse events [57]. Development of antibodies against transfused blood products negates their therapeutic use, requiring challenging treatment alternatives [58,59]. To investigate the translational potential of dPLT-mediated immunosuppression in transfusion medicine, we first tested whether desialylated human platelets possess immunosuppressive effects against an independent secondary challenge of sRBCs. We found high-dose presensitization with human dPLT significantly decreased anti-sRBC titers following challenge with one dose of sRBC (Fig. 7A).

We next honed on the disease hemophilia A, a common congenital bleeding disorder due to absence of FVIII. Replacement FVIII therapy frequently leads to detrimental anti-FVIII inhibitory antibody generation [58]. We utilized a murine model of hemophilia A (FVIII^null^), which is highly prone to develop anti-FVIII immune responses upon rhFVIII infusion, recapitulating human disease [60–65]. We previously demonstrated that anti-FVIII inhibitory antibodies could be prevented in FVIII^null^ when transfused FVIII was carried by FVIII-expressing transgenic platelets (2bF8^Tg^) [60,62]. However, transfusion of 2bF8^Tg^ failed to protect FVIII^null^ mice against a subsequent anti-FVIII response when challenged with rhFVIII [60]. To investigate whether desialylated 2bF8^Tg^ platelets could attenuate antibody response following secondary challenges of rhFVIII, we adopted a similar sensitization strategy (Fig. 7B and C). None of the mice developed anti-FVIII inhibitors after 4 doses of dPLTs infusion (Fig. 7D). Importantly, anti-FVIII inhibitory titers in the 2bF8^Tg^ dPLT-presensitized group was significantly lower than in the control group without 2bF8^Tg^ dPLT (130 ± 154 Bethesda unit (BU)/ml versus 343 ± 184 BU/ml; Fig. 7D). Although 4 of 9 presensitized FVIII^null^ mice developed high titers (>100 BU/ml) of anti-FVIII inhibitors following rhFVIII immunization (Fig. 7E), of the remaining 5 mice, 3 did not develop anti-FVIII inhibitors, and the other 2 had low titers (2.9 and 13 BU/ml, respectively). In contrast, all control FVIII^null^ mice without dPLT presensitization developed anti-FVIII inhibitors when immunized with the same immunization protocol, and 93% of animals developed greater than 100 BU/ml of anti-FVIII inhibitors, which is significantly higher than the 2bF8^Tg^ dPLT-presensitized group (*P* < 0.01) (Fig. 7E). These results demonstrate that dPLT presensitization can attenuate alloimmune or isoimmune responses during blood transfusions, with potential therapeutic value.

## Discussion

The liver is constantly exposed to environmental neoantigens and microbial and food antigens from the intestine, necessitating an immunotolerant milieu to prevent hyperimmune activation [55,66]. Under normal conditions, antigens presented within this niche generate a blunted immune response locally and have been shown to induce tolerance systemically [67]. We identify that near-exclusive clearance of dPLT in the liver, which given the large vascular beds within the lung and spleen, indicates active platelet capture and not passive endothelial adherence. Previously, it was widely accepted dPLT clearance mediated by hepatocytes via the AMR predominates [21]. However, the restrictive size limitations of the endothelial fenestrae and uptake capacity of hepatocytes suggest other more likely candidates [68,69]. Consistent with recent emerging reports [39,41], we identify macrophages as the primary cell mediating dPLT clearance as evidenced by the rescue of thrombocytopenia in the presence of clodronate depletion of macrophages, but not AMR deficiency. As clodronate liposomes deplete not only Kupffer cells but also splenic macrophages, we cannot exclude the phagocytic contribution from splenic macrophages. However, we did not observe a significant difference in clearance kinetics of dPLT in splenectomized mice, suggesting a minor role. Furthermore, in our in vitro phagocytic assays, where to the best of our knowledge, we are the first to demonstrate that splenic and monocyte-derived macrophages are less proficient at dPLT clearance compared to Kupffer cells.

The lack of in vivo dPLT targeting to splenic macrophages is intriguing. Marginal zone and red pulp macrophages in the spleen come into direct contact with dPLT in circulation. However, it is unknown whether they express the required lectin receptors to recognize exposed underlying desialylated galactose residues [70,71]. In addition, expression of canonical inhibitory phagocytic receptor signal regulatory protein α (SIRPα), not expressed on Kupffer cells, may cumulatively contribute to the observed functional dichotomy and net effect of decreased dPLT uptake by splenic macrophages [72,73].

We did not observe a significant contribution of either AMR or platelet GPIbα for hepatic targeting, as most dPLT maintained hepatic clearance in Ashwell–Morell-deficient mice or with GPIbα-deficient platelets. Interestingly, we did observe increased lung targeting of desialylated GPIbα-deficient platelets. Given that lung macrophages are located within the subendothelial space, the increased sequestration may be due to increased interactions and tethering to the endothelium, rather than macrophage capture, following desialylation in the absence of GPIbα [74]. It has been reported that GPIbα blocking increases seeding and lung metastasis of cancer cells; whether it is due to increased platelet targeting to the lung is unknown; thus, further studies are warranted to investigate the exact role of GPIbα in lung recruitment of platelets [75].

Recent reports have identified some putative ligand–receptor partners for Kupffer cell-mediated dPLT uptake [39,41]. We observed a synergistic requirement of GPIbα and AMR for hepatic targeting and macrophage association. In the absence of both, the sequestration of dPLT was diffuse and found in large amounts in both the spleen and liver, where associations with Kupffer cells significantly decreased. We hypothesize that GPIbα contributes to dPLT adherence and tethering in the liver and contributes to Kupffer cell-mediated uptake. This is a similar phenotype to a recent report of synergistic blocking of hepatic AMR and Kupffer macrophage galactose-type lectin receptor (MGL) [39]. Thus, it is likely that the ligand for MGL may be platelet GPIbα or MGL may indirectly interact with GPIbα via von Willebrand factor (VWF), although further investigation is warranted.

Interestingly, we observed that antibody opsonization of dPLT did not alter the predominant hepatic targeting but did alter the macrophage subtype, switching from immunosuppressive M2 Kupffer cells to M1-proinflammatory monocyte-derived M1 macrophages [17]. Phenotypic heterogeneity of macrophages within the liver has been reported to exist within a spatial gradient, with a highly immunosuppressive polarized population localized closest to the periportal triad [76,77]. Whether antibody-opsonized dPLTs are cleared by resident M1-like Kupffer cells or via monocytes recruited from the blood circulation is unknown. It has been previously reported that increased numbers and activity of M1 proinflammatory macrophages within the liver abrogate the immunotolerant milieu of the liver; thus, whether immune-mediated thrombocytopenias skew the liver toward proinflammation deserves further investigation [78].

Kupffer cells have been reported to contribute to the induction and maintenance of systemic tolerance of 54. At baseline, Kupffer cell antigen uptake produces the anti-inflammatory cytokines IL-10 [78] and TGF-β [79]. We demonstrate that uptake of dPLT further increases these anti-inflammatory cytokines and may contribute to the maintenance of circulating CD4^+^ T_regs_ observed. This may be the basis of the correction and elevation of CD4^+^ T_reg_ levels as seen in immune thrombocytopenic patients who experience normalization of platelet numbers [80,81]. Similarly, we observed an increased number and function of CD4^+^ T_regs_ following increasing circulating dPLTs. Notably, non-NEU-treated WT platelet transfusion also induced increased CD4^+^ T_reg_ numbers; we attribute this to eventual senescence and desialylation at late stage for both exogenous and endogenous platelets and subsequent hepatic clearance [39], which may contribute to a general immunosuppressive state restricting autoimmunity. Clinically, a general correlation between platelet mass and increased immunosuppression has been long observed, such as in transfusion-mediated immune suppression and increased incidence of infections and cancer following allogeneic platelet transfusions [82]. More recently, thrombopoietin (TPO) mimetic therapy in immune thrombocytopenic patients saw ~30% of patients who experience long-term remission following the tapering of the drug, which has been linked to decreased antibody generation and correction of immune dysregulation [80,83,84].

The mechanism of function of the increased CD4^+^ T_regs_ is currently unknown. However, it is at least partially contributory to the suppression of the adaptive immune response that we observed in an antigenic challenge, as seen with our adoptive transfer data. The immunosuppression may be antigen nonspecific as we observed here and in our earlier study with other T_regs_ [85], although the suppressive effect was more robust with platelet-associated antigens. Further studies are warranted to elucidate the limits of the immunosuppression, including the duration of the effector-suppressive function.

The findings here indicate that platelet-associated antigens could induce an immunosuppressive state through Kupffer cells, which, we demonstrate, carries through in relevant clinical transfusion models. This also introduces therapeutic potential of dPLT transfusions to promote immunosuppression or to utilize dPLT as delivery vehicles for antigens requiring cover from immune surveillance, such as replacement FVIII in hemophilia A. Furthermore, in platelet transfusions, dPLT or its derivatives, previously nonutilized because of decreased circulating lifespan, may now have therapeutic utility.

As platelets are known scavengers of the circulatory system, it is conceivable that clearance of senescent platelets requires an immunotolerant response leading to clearance in the liver. Conversely, increased dPLT hepatic targeting of detrimental antigens such as cancer aggregates or viral particles may protect them from immune targeting. Further studies are required to explore the full implications of this novel immunosuppressive pathway within different diseases [86–90].

## Materials and Methods

### Mice

BALB/c, C57BL/6, and splenectomized BALB/c mice were purchased from Charles River Canada. IL-4Rα/GPIbα-tg [91], GPIb^−/−^ [92], β3^−/−^ [93], and *Asgr2*^−/−^ [94] mice were gifts from J. Ware, Z. M. Ruggeri, R. O. Hynes, and K. Hoffmeister, respectively. FVIII-deficient (FVIII^null^) mice were a gift from H. Kazazian. 2bF8^Tg^ mice, which are transgenic animals with platelet-specific human B domain-deleted FVIII expression, were generated by lentiviral transgenesis as previously reported [60,95]. All mice were used between 8 and 16 weeks for experiments. All animal studies were approved by the Institutional Animal Care and Use Committee of either the St. Michael’s Hospital, Canada, or the Medical College of Wisconsin, USA.

### Blood collection and platelet isolation

Procedures were approved by the Research Ethics Board of St Michael’s Hospital (Toronto, ON, Canada) and conducted as previously described [22]. Venous blood was obtained from healthy volunteers by venipuncture into 3.2% trisodium citrate. Platelet-rich plasma (PRP) was prepared by centrifugation (10 min, 300*g*, no brake, 22 °C). Platelets were isolated from PRP and washed (15 min, 1,050*g*, no brake, 22 °C, with prostaglandin I2 of 10 ng ml^−1^) and resuspended in buffer B [10 mM Hepes, 140 mM NaCl, 3 mM KCl, 0.5 mM MgCl_2_, 10 mM glucose, and 0.5 mM NaHCO_3_ (pH 7.4)]. Mice were bled via retro-orbital bleeding into anticoagulant citrate dextrose. PRP was obtained with centrifugation (250*g*, 7 min, no brake). Washed platelets was then obtained from PRP with centrifugation (800*g*, 8 min, no brake) and resuspended in buffer B.

### Immunization and antibody titration

Indicated mice were immunized with indicated platelets or washed sRBCs (Colorado Serum Company) via tail vein (or otherwise indicated) at the indicated dose, weekly for the indicated number of weeks. Sera was then collected via saphenous vein and serially diluted (1:2) in phosphate-buffered saline (PBS) and incubated with 2 × 10^6^ platelets of the genetic background of the most recent immunization for 1 h at room temperature. Samples were then washed (800*g*, 8 min, no brake) and incubated with 1:1,000 dilution F(ab′)_2_ goat anti-mouse IgG (H+L) Alexa Fluor 647 secondary antibody (Thermo Fisher Scientific) for 30 min at room temperature and read by flow cytometry.

### Ex vivo fluorescent labeling and desialylation of platelets

5-chloromethylfluorescein diacetate (5 μM) or CellTracker Far Red (Thermo Fisher Scientific) was added to 2 × 10^8^/ml of washed platelets in Pipes buffer to fluorescently label platelets. NEU (2.5 mU; EMD4) was sometimes added to dPLTs. Platelet preparation was then incubated in 37 °C water bath for 45 min followed by a 5× dilution with buffer B, and washed 2× (800*g*, 8 min, no brake) in the presence of 10 μM prostacyclin (Cayman Chemical). Desialylation was checked with 1:1,000 dilution of fluorescein-labeled Ricinus Communis Agglutinin I (Vector Laboratories). Samples were read by flow cytometry on a BD LSRFortessa X-20.

### In vivo platelet circulation studies

Platelet membranes were fluorescently labeled and sometimes desialylated with NEU as described earlier. A total of 10^8^ labeled platelets were transfused via tail vein into syngeneic mice. Mice were bled at 1, 15, and 30 min and 16 h after transfusion via saphenous bleed into PBS-EDTA. PRP was tested with flow cytometry to assess percentage dye^+^ platelets. To induce in vivo platelet desialylation, mice were injected intraperitoneally with NEU (2.5 mU/g) or mouse anti-mouse αIIb (0.05μg/g) in-house-generated monoclonal antibody [22]. Platelet counts on indicated days were taken as described above. In some cases, Kupffer cell depletion was preformed 2 days prior to NEU injection with intravenous injection of 0.01 ml/g of body weight of clodronate liposome or control liposome (Liposoma).

### ICG labeling of platelets

ICG (3 μg/ml; Sigma-Aldrich) was incubated with 10^8^ platelets in PBS-EDTA on a rotator for 1 h to label platelets. Labeled platelets were washed 2× (800*g*, 8 min, no brake) and resuspended in buffer B. Successful labeling of platelets was checked on a Molecular Devices m5e multimode plate reader on absorbance on wavelengths spanning from 600 to 900 nm. Data were analyzed on SpectraMax software.

### Multispectral optoacoustic imaging

Agar phantoms with ICG-labeled platelets were scanned as previously described [96]. Prior to imaging, the torso regions of mice were gently shaved with a hair trimmer (ChroMini, WAHL, IL) under general isofluorane anesthesia. Hair removal cream (Nair, Church & Dwight, ON, Canada) was applied for 30 s to remove remaining hair and thin layer of clear ultrasound gel (Aquasonic Clear, Parker Laboratories, NJ), warmed using a warmer (Thermasonic Gel Warmer, Parker Laboratories), and was applied. Depilated mice under general anesthesia were placed in the MSOT (MSOT 128, iThera Medical GmbH, Munich, Germany) machine with a tail–vein catheter, to allow for baseline scans prior to platelet transfusion without disturbing body placement. Washed dPLTs labeled with ICG (3 μg/ml), or control ICG-labeled platelets, were transfused after acquiring background scans. Scans were acquired at wavelengths between 680 and 900 nm every 5 min at multiple slices for 1 h following platelet transfusion. Data were collected and analyzed using the native software (ViewMSOT, iThera Medical GmbH) in conjunction with iThera support.

### Flow cytometry analysis of tissue distribution of labeled transfused platelets

Washed desialylated platelets or control-labeled platelets were injected intravenously. Mice were bled and sacrificed at indicated time points after transfusion. The organs were harvested and mashed through a 50 μM filter and fixed and RBCs lysed in 1× Fix/Lyse buffer (BD). Cells were washed (400*g*, 5 min) and resuspended in flow staining buffer [PBS/2% bovine serum albumin (BSA)/0.01% sodium azide], and Fcγ receptors blocked with anti-CD16/32 (10 μg/ml; 20 min) on ice. Samples were then stained with phycoerythrin-anti-CD41 (MWreg30) (0.2μg/ml; BioLegend) for 45 min on ice, before washing (400*g*, 5 min) and analyzing via flow cytometry.

### Immunohistochemistry

Washed dPLTs or control-labeled platelets were injected intravenously. Mice were bled and sacrificed at 2 h after transfusion. Liver was perfused with Liver perfusion medium (Thermo Fisher Scientific) through the hepatic portal vein, and lungs were perfused with optimal cutting temperature (Thermo Fisher Scientific) compound through the trachea before harvest and snap-frozen in liquid nitrogen. Frozen tissue sections (5 μM) on slides were fixed with 4% paraformaldehyde (PFA) for 15 min. Sections were blocked with PBS-Tween 20 + 2% BSA and 5% goat serum for 20 min at room temperature, before staining overnight at 4 °C with anti-F480 (5 μg/ml; clone BM8) and anti-αIIb (in-house generated mouse anti-mouse, clone 5C4). Secondary staining was performed with anti-mouse 488 and anti-rat Cy3 in 1:500 dilution for 2 h at room temperature and 4′,6-diamidino-2-phenylindole (DAPI) staining at 1:12,000 dilution for 5 min. We mounted with DAKO mounting media overnight, imaged on Zeiss Axioscan Z1, and analyzed with HALO (Indica Labs) software.

### In vitro phagocytosis assay

Spleen and livers from anesthetized mice were harvested following liver perfusion with liver perfusion media through cannulation of portal vein and mashed through 70 μM cell strainer to generate single-cell suspensions. Red blood cell lysis was performed with ACK. Liver parenchymal cells were precleared with centrifugation at 50*g* for 3 min. Remaining cells were gently layered on 15 ml of lymphoprep (STEMCELL technologies) and subject to density centrifugation at 800*g* for 25 min. Lymphocyte interphase was washed 3× before counting. Liver cells and splenocytes were plated at 10^6^ cells in 12-well plates. THP-1 cells were plated at 10^6^ cells in 12-well plates and differentiated overnight with phorbol 12-myristate 13-acetate (50 ng/ml). A total of 5 × 10^5^ RAW264.7 cells were platelet in 12-well plates on coverslips in RPMI 1640, 10% fetal bovine serum, and 1% penicillin/streptomycin for 48 h in a 37 °C and 5% CO_2_ humidified chamber. Desialylated labeled platelets, control-labeled platelets, or labeled platelets incubated with antiplatelet antibody 9D2 or 5C4 (2 μg/10^8^) were added to the wells and incubated for 2 h. Wells were washed 3× with Pipes and incubated with 1:1,000 dilution of fixable viability dye Zombie yellow. Following which cells were gently scraped and fixed with 4% PFA at room temperature for 15 min, fixed cells were washed in flow staining buffer (PBS, 2 mM EDTA, 2% BSA, and 0.1% sodium azide), blocked with FcBlock (10 μg/ml; BD) followed by staining with anti-F4/80 (BM8) (0.5 μg/ml; eBioscience) for 45 min at room temperature, and analyzed with flow cytometry. In other wells, 4% PFA was added after anti-F4/80 staining, and coverslips were removed from wells and mounted on glass slides in Prolong Diamond Antifade Mountant (Thermo Fisher Scientific) before visualizing on a Zeiss LSM700 confocal.

### In vitro cytokine production

Single-cell liver suspensions and parenchymal preclearance were prepared as described above. Kupffer cells were isolated at the interphase of 25% and 50% Percoll gradient solution following density centrifugation at 1,350*g* for 30 min. Cells were washed and plated at a density of 10^6^ viable cells in 24-well plates. Plates were washed at 4 h post to remove nonadherent cells. A total of 5 × 10^6^ WT or dPLTs and LPS O111:B4 (1 μg/ml; Sigma-Aldrich) were added to the wells and incubated for indicated time points. Golgi stop (1,000×) dilution was added 6 h before harvest. Cells were harvested by gentle scraping and were stained with fixable viability stain Zombie Aqua (BioLegend) followed by fixation with 4% PFA and blocked with FcBlock (10 μg/ml; BD) prior to staining for flow cytometry. Cells were permeabilized for intracellular staining with 1× perm wash buffer (BioLegend) according to the manufacturer’s recommendations.

### PBMC immunophenotyping

Mice that were transfused intravenously with 2 × 10^8^ desialylated or control platelets were bled via saphenous vein into BD Vacutainor EDTA tubes. Whole blood was fixed, and RBCs lysed with 1× Fix/Lyse buffer (BD) for 30 min at room temperature and washed 2× in flow staining buffer (400*g*, 5 min). PBMC were then stained with anti-CD4^+^ (GK1.5) (0.25 μg/ml; eBioscience), anti-CD3 (17A1; 0.5 μg/ml), and anti-CD25 (PC61.5; 0.3 μg/ml) in one tube for CD4^+^ T_regs_. CD3^+^CD4^+^CD25^+^ tube was washed and resuspended in permeabilization buffer (PBS/0.1% saponin/2% BSA) for 20 min at room temperature before adding anti-FoxP3 (150D; 0.75 μg/ml) and incubating for 1 hour at room temperature. Samples were washed an analyzed by flow cytometry.

### T_reg_ suppression assay

Spleens were harvested from GPIb^−/−^ mice transfused 2× with either 10^8^ WT or desialylated BALB/c platelets. Single-cell suspensions were prepared as described above, and CD4^+^CD25^+^ T_regs_ and CD4^+^CD25^^−^^ conventional T cells were cell sorted to >95% purity on an FACSAria III. Conventional T cells were stained with 5 μM carboxyfluorescein diacetate succinimidyl ester and resuspended in RPMI 1640 complete. A total of 2.5 × 10^5^ cells per well were plated in 96-well plates. T_regs_ were added in 2× serial dilutions starting with 1.25 × 10^5^. The ratio (1:1) of Dynabeads (Thermo Fisher Scientific) was also added to stimulate T cell proliferation. After 5 days, cells were harvested and prepared for flow cytometry as described above.

### Hemophilia A mouse model

FVIII-deficient (FVIII^null^) mice were generated via a targeted disruption of exon 17 of the *F8* gene with undetectable FVIII in the plasma [65], and the colony was maintained in our facility. 2bF8 mice (2bF8^Tg^) are transgenic with platelet-specific human B domain-deleted FVIII expression generated by lentiviral transgenesis [60,95]. Both FVIII^null^ and 2bF8^Tg^ mice were on C57BL/6 and 129S mixed genetic background. All mice were maintained in pathogen-free microisolator cages at the animal facilities operated by the Medical College of Wisconsin. Animal studies were performed according to protocols approved by the Institutional Animal Care and Use Committee of the Medical College of Wisconsin. Isoflurane or ketamine was used for anesthesia.

Platelets were isolated from 2bF8^Tg^ mice as previously described [60,61] and treated with α2-3, 6, 8, and 9-NEU (10 mU/ml) in modified Tyrode buffer for 5 to 6 h. Platelets were then washed and resuspended in Tyrode buffer with a concentration of (1.5 to 3.0) × 10^9^ platelets/ml. To determine the percentage of platelets that were desialylated, 1 × 10^6^ of NEU-treated platelets were stained with Fluorescein Ricinus Communis Agglutinin I (Vector Laboratories Inc., Burlingame, CA) and analyzed by flow cytometry. dPLTs in Tyrode buffer (10 μl/g of body weight) were infused into FVIII^null^ mice via retro-orbital venous administration weekly for 4 weeks. One week after the last dPLT infusion, blood samples were collected, and plasma was isolated for Bethesda assay as we previously reported [62] to determine anti-FVIII inhibitory antibodies titers (referred to as inhibitors). Animals were further immunized with recombinant human B domain-deleted FVIII (rhFVIII, Xyntha; Pfizer Inc., NewYork, NY) at a dose of 50 U/kg per week for 4 weeks through retro-orbital venous administration. One week after the last rhFVIII immunization, blood samples were collected, and inhibitor titers were determined.

### Statistical analysis

Unless noted otherwise, a 2-tailed, unpaired *t* test was used to assess statistical significance. Statistical calculations were performed in GraphPad Prism 7. The number of replicates and a description of the statistical method are provided in the corresponding figure legends. Differences with *P* values of less than 0.05 were considered to be statistically significant. *P < 0.05, **P < 0.01, and ***P < 0.001; ns indicates not significant.

## Data Availability

All data needed to evaluate the conclusions in the paper are present in the paper or the Supplementary Materials.

## Supplementary Materials

Supplementary 1 Fig. S1. No significant loss of surface expression of GPIbα and GPIIbIIIa following NEU treatment. Fig. S2. Desialylated sRBCs do not induce lower antibody generation. Fig. S3. dPLTs do not induce significant differences in antibody repertoire compared with WT platelets. Fig. S4. Absorbance spectrum of ICG-labeled platelets in 10-nm increments from 600 to 900 nm yields a characteristic curve. Fig. S5. Intravital microscopy of mesenteric vessels reveals stable adhesion of dPLT but not WT on vessel walls. Fig. S6. Flow cytometry analysis of small intestine shows increased amounts of transfused dPLT. Fig. S7. Whole tissue confocal imaging of small intestine shows dPLT localization in the lamina propria. Fig. S8. Clodronate treatment significantly depletes CD11b^+^F4/80^+^ macrophages in the spleen and liver. Fig. S9. No significant increases in other proinflammatory cytokines interferon-γ and IL-2 production by F4/80^+^ Kupffer cells in presence of different platelet coincubations. Fig. S10. NEU and anti-αIIb monoclonal antibody treatment causes similar kinetics of thrombocytopenia.
