# Supplementary material for: Desialylated Platelet Clearance in the Liver is a Novel Mechanism of Systemic Immunosuppression
Source: Research (Wash D C). 2023 Oct 5;6:0236. doi: 10.34133/research.0236 (PMC10551749; doi:10.34133/research.0236)
Supplement: Supplementary 1 — Fig. S1. No significant loss of surface expression of GPIbα and GPIIbIIIa following NEU treatment. Fig. S2. Desialylated sRBCs do not induce lower antibody generation. Fig. S3. dPLTs do not induce significant differences in antibody repertoire compared with WT platelets. Fig. S4. Absorbance spectrum of ICG-labeled platelets in 10-nm increments from 600 to 900 nm yields a characteristic curve. Fig. S5. Intravital microscopy of mesenteric vessels reveals stable adhesion of dPLT but not WT on vessel walls. Fig. S6. Flow cytometry analysis of small intestine shows increased amounts of transfused dPLT. Fig. S7. Whole tissue confocal imaging of small intestine shows dPLT localization in the lamina propria. Fig. S8. Clodronate treatment significantly depletes CD11b+F4/80+ macrophages in the spleen and liver. Fig. S9. No significant increases in other proinflammatory cytokines interferon-γ and IL-2 production by F4/80+ Kupffer cells in presence of different platelet coincubations. Fig. S10. NEU and anti-αIIb monoclonal antibody treatment causes similar kinetics of thrombocytopenia. [file research.0236.f1.docx]

**Supplementary figures**

**Desialylated platelet clearance in the liver**

**is a novel mechanism of systemic immunosuppression**

June Li, Danielle Karakas, Feng Xue, Yingyu Chen, Guangheng Zhu, Yeni H. Yucel, Sonya A MacParland, Haibo Zhang, John W. Semple, John Freedman, Qizhen Shi^*^ and Heyu Ni^*^


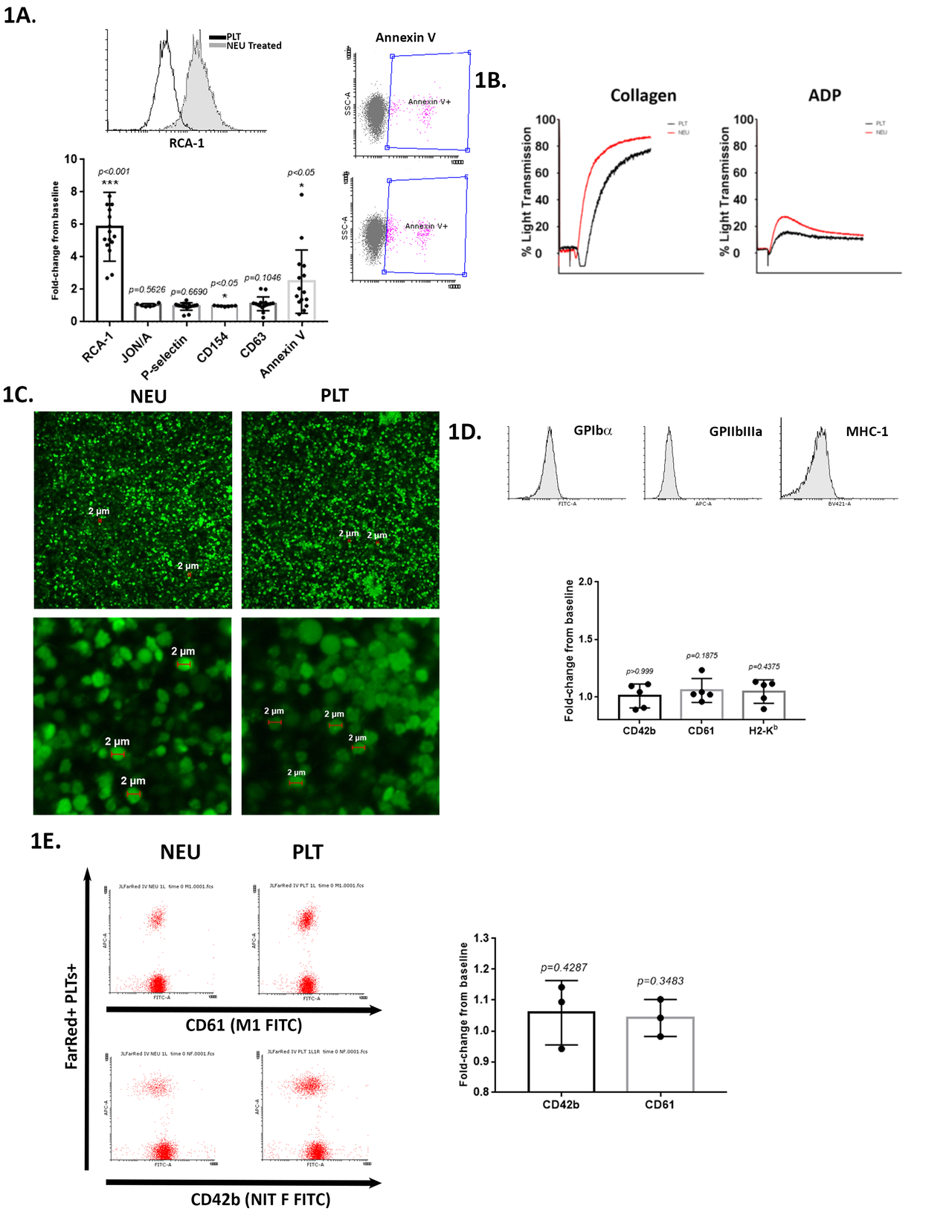


**Supplementary Figure 1.** No significant loss of surface expression of GPIbα and GPIIbIIIa following Neuraminidase treatment as detected by flow cytometry


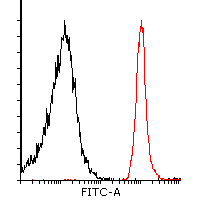


**NEU sRBC**

**sRBC**

**RCA-1**

**Supplementary Figure 2.** Desialylated sheep red blood cells (sRBC) do not induce lower antibody generation. Representative histogram demonstrating significant desialylation of sRBC by Neuraminidase (NEU) treatment. Data represented as mean±SD

Anti-GPIbα

Anti-GPIIbIIIa

**Supplementary Figure 3.** Desialylated platelets does not induce significant differences in antibody repertoire compared with WT platelets. (a) Anti-GPIbα and (b) anti-GPIIbIIIa antibodies titers as detected binding to desialylated (NEU) or WT platelets. All data represented as mean±SD. ****p>0.001, *p>0.05

 
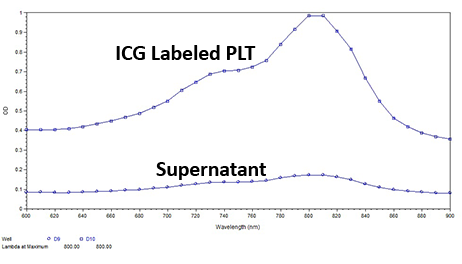


**Supplementary Figure 4.** Absorbance spectrum of ICG labeled platelets in 10nm increments from 600 to 900nm yields a characteristic curve, with distinct peak absorbance at ~800nm indicating successful labeling. Negligible absorbance was detected in the incubation supernatant following washing indicating low ICG decoupling and stable labeling.


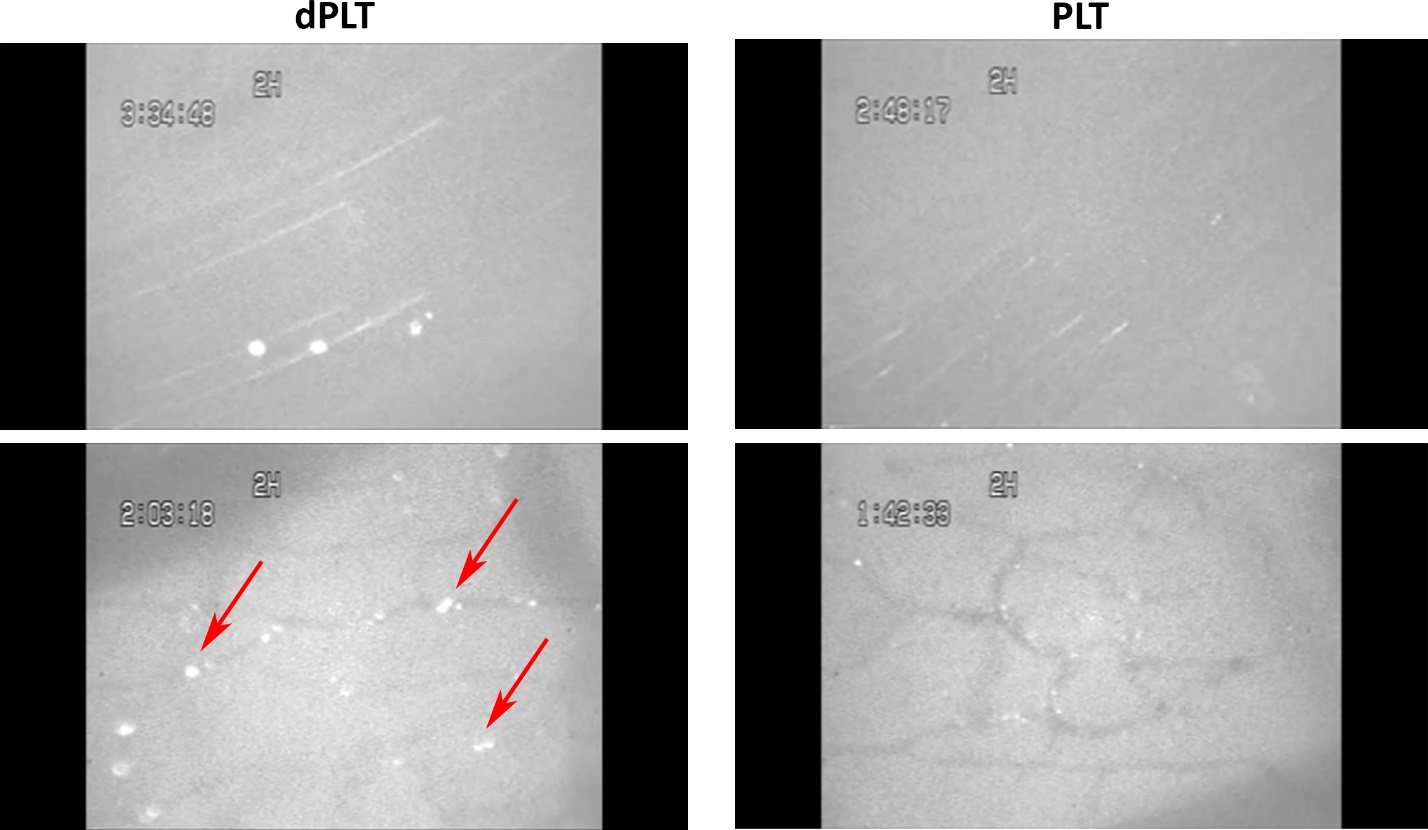


**Supplementary Figure 5.** Intravital microscopy for murine mesenteric vasculature reveal stable adherence of dPLT in the vein and capillaries (red arrows) not observed with WT PLTs. Representative images from n=3 mice. Imaging of mesenteric vasculature was performed as previously described ^1^, without application of Ferric Chloride injury.


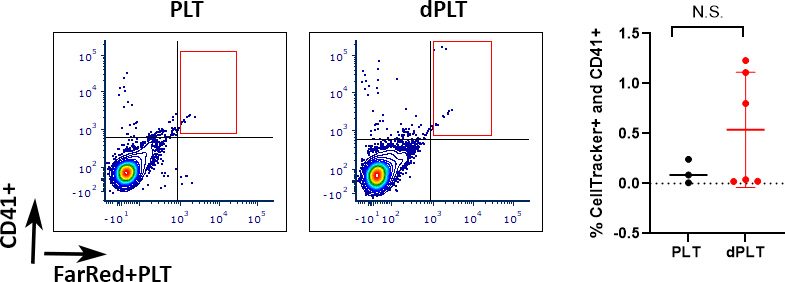


**Supplementary Figure 6.** Flow cytometry analysis of single cell suspension of small intestine harvested 2 hours post FarRed+PLT transfusion reveal trend towards increased presence of dPLT. A total of 50,000 events were acquired.


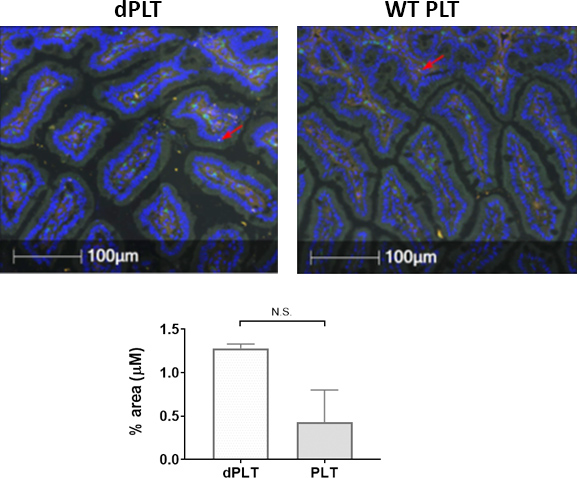


**Supplementary Figure 7.** Confocal tissue sections of small intestine reveal a trend of increased dPLT localization in the lamina propria of small intestine (Red Arrows). Green is F4/80 macrophage, Orange is FarRed+PLTs. Blue is DAPI. Data quantified as % of area positive for FarRed+PLTs+ in whole tissue slides, as assessed with HALO software (Indica Labs).


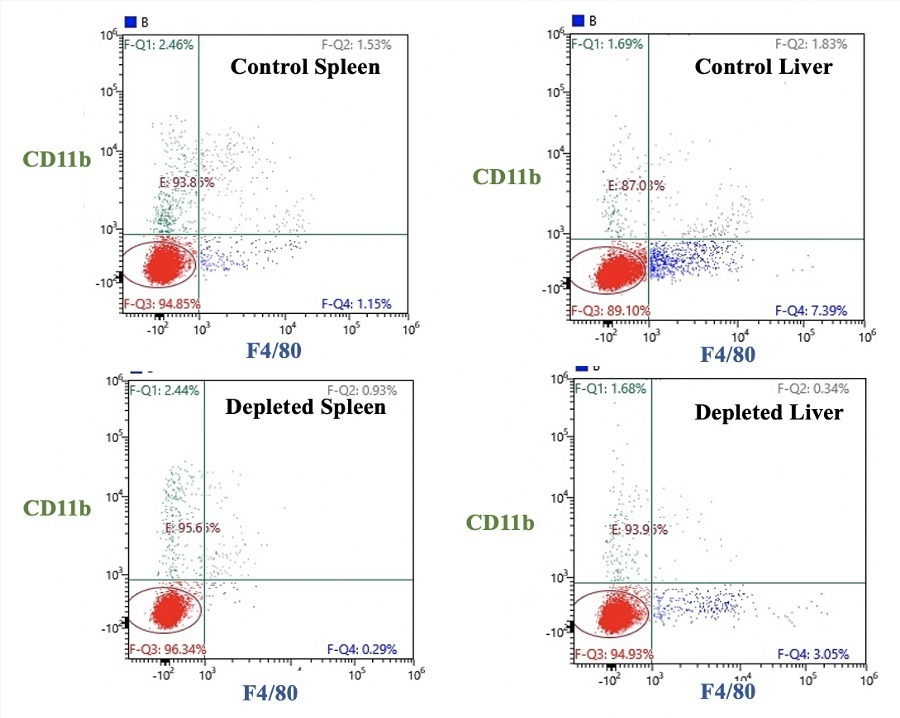

**Supplementary Figure 8.** Clodronate treatment significantly depletes CD11b+F4/80+ Macrophages in spleen and liver. Representative dot plots of spleen and liver on day 2 following clodronate liposome depletion via 0.01mg/g body weight intravenous injection.

**
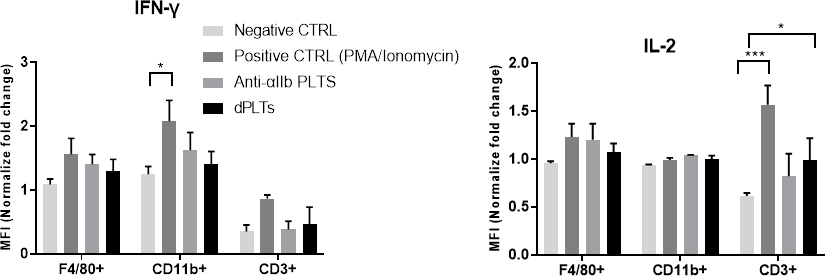
 Supplementary Figure 9.** No significant increases in other pro-inflammatory cytokines IFN-γ and IL-2 production by F4/80+ Kupffer cells in presence of different platelet co-incubations. Data represented as fold change over isotype control, and as SEM. N=5-8. ***p>0.001, *p>0.05


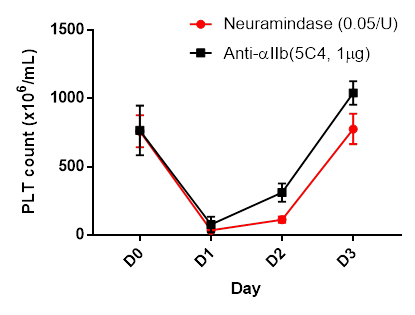


**Supplementary Figure 10**. Neuraminidase and anti-αIIb monoclonal antibody cause similar kinetics of thrombocytopenia. WT BALB/c mice were intravenously transfused with indicated dose of Neuraminidase or anti-αIIb mouse anti-mouse monoclonal antibody. Platelet (PLT) counts were measured on days 1, 2, and 3. N=5

References:

1. Ni, H.*, et al.* Persistence of platelet thrombus formation in arterioles of mice lacking both von Willebrand factor and fibrinogen. *J Clin Invest* **106**, 385-392 (2000).
